# Metabolite Profiling of *adh1* Mutant Response to Cold Stress in *Arabidopsis*

**DOI:** 10.3389/fpls.2016.02072

**Published:** 2017-01-11

**Authors:** Yuan Song, Lijun Liu, Yunzhu Wei, Gaopeng Li, Xiule Yue, Lizhe An

**Affiliations:** Ministry of Education Key Laboratory of Cell Activities and Stress Adaptations, School of Life Sciences, Lanzhou UniversityLanzhou, China

**Keywords:** metabolite profiling, *adh1* mutant, freezing stress without cold acclimation, freezing stress with cold acclimation, *Arabidopsis*

## Abstract

As a result of global warming, vegetation suffers from repeated freeze-thaw cycles caused by more frequent short-term low temperatures induced by hail, snow, or night frost. Therefore, short-term freezing stress of plants should be investigated particularly in light of the current climatic conditions. Alcohol dehydrogenase (ADH) plays a central role in the metabolism of alcohols and aldehydes and it is a key enzyme in anaerobic fermentation. ADH1 responds to plant growth and environmental stress; however, the function of ADH1 in the response to short-term freezing stress remains unknown. Using real-time quantitative fluorescence PCR, the expression level of *ADH1* was analyzed at low temperature (4°C). The lethal temperature was calculated based on the electrolyte leakage tests for both *ADH1* deletion mutants (*adh1*) and wild type (WT) plants. To further investigate the relationship between *ADH1* and cold tolerance in plants, low-Mr polar metabolite analyses of *Arabidopsis adh1* and WT were performed at cold temperatures using gas chromatography-mass spectrometry. This investigation focused on freezing treatments (cold acclimation group: −6°C for 2 h with prior 4°C for 7 d, cold shock group: −6°C for 2 h without cold acclimation) and recovery (23°C for 24 h) with respect to seedling growth at optimum temperature. The experimental results revealed a significant increase in *ADH1* expression during low temperature treatment (4°C) and at a higher lethal temperature in *adh1* compared to that in the WT. Retention time indices and specific mass fragments were used to monitor 263 variables and annotate 78 identified metabolites. From these analyses, differences in the degree of metabolite accumulation between *adh1* and WT were detected, including soluble sugars (e.g., sucrose) and amino acids (e.g., asparagine). In addition, the correlation-based network analysis highlighted some metabolites, e.g., melibiose, fumaric acid, succinic acid, glycolic acid, and xylose, which enhanced connectedness in *adh1* network under cold chock. When considered collectively, the results showed that *adh1* possessed a metabolic response to freezing stress and *ADH1* played an important role in the cold stress response of a plant. These results expands our understanding of the short-term freeze response of *ADH1* in plants.

## Introduction

Freezing shock (<0°C) due to hail, snow, or night frost is a potentially lethal stress for plants. Freezing tolerance is a central factor influencing yield and geographical distribution of crops. The mechanisms of the tolerance capability differ between sudden freezing shock and freezing stress with prior cold acclimation. Sudden freezing shock induces cell membrane damage and osmotic dehydration of cells via the growing of extracellular ice crystals. However, cold acclimation has been reported to improve cold tolerance in rice via intermediated physical, chemical, and metabolic processes, i.e., cold acclimation processes enhanced the expression of root-specific aquaporin to increase water uptake (Ahamed et al., 2012). Cold acclimation is a strategy that plants use to cope with the primary cause of chilling injury (Joe, 1978). Cold acclimation may impede a phase transition at freezing stress. Furthermore, the increase of C1 to C9 alcohols lowers the phase transition of cell membranes, enabling plants to better cope with a sudden drop in temperatures (Thomashow, 1999). Alcohols enhance membrane fluidity, thus avoiding the phase transition of cellular membranes from liquid to gel that happens in chilling and freezing injuries (Ingram and Buttke, 1984). Among the numerous cold-responsive genes, *ADH1* can produce C1 to C9 alcohols to protect plant cells from freezing damage.

The classic alcohol dehydrogenase (ADH, alcohol: NAD+ oxidoreductase, EC 1.1.1.1) is a Zn-binding enzyme that acts as a dimer and relies on a NAD(P) co-factor to interconvert ethanol and acetaldehyde (as well as other short linear alcohol/aldehyde pairs) (Strommer, 2011). ADH activity is subject to various stresses (Dolferus et al., 1994), including biotic (Pathuri et al., 2011), and abiotic stress (Komatsu et al., 2011). However, the functional mechanism is not completely clear. ADH is related to the fermentative metabolism reduces acetaldehyde to ethanol, regenerates NAD+ and 2 ATP, and protects cells from cytoplasmic acidosis (Drew, 1997). Ethanol enhances ATPase synthesis in different species (Lalitha et al., 1987, 1988; Monteiro and Sá-Correia, 1998; Hu et al., 2004). ATPase-catalyzed proton pumping may contribute to chilling tolerance in plants (Löw et al., 1996; Sze et al., 2002). ADH1 plays a key role in maintaining the stability of the membrane structure for the enhancement of cold resistance in plants.

Due to the importance of ADH, numerous reports have recently been conducted about the function of ADH. Furthermore, plant *ADH* genes play a long-standing role in evolutionary studies (Strommer, 2011) and the structure of ADH1 in *Arabidopsis thaliana* was parsed (Cheng et al., 2013; Chen et al., 2015). Previous studies have found a close relationship between ADH and plant metabolites. Metabolomic studies revealed ADH to play a central role in the biosynthesis of an important group of aroma volatiles (C6-derivative compounds) including different aldehydes, alcohols, and esters (Bicsak et al., 1982; Molina et al., 1986; Longhurst et al., 1990; Millán et al., 1990; Speirs et al., 1998). These metabolites are related to fruit flavors, ripening of fruits, antiviral resistance, environmental stresses responses and the ABA phytohormone (Dolferus et al., 1994; Zhang et al., 1997). The *ADH1* gene mutant of *Chlamydomonas reinhardtii* elicits metabolic restructuring during dark anoxic growth (Catalanotti et al., 2012; Magneschi et al., 2012). Exploring the temperature-induced change of the metabolome is a current field of interest (Kaplan et al., 2004; Sun et al., 2016), including metabolite composition of freezing tolerance (Korn et al., 2010). The results revealed that pyruvate, oxaloacetate, polyamine precursors, and compatible solutes increased during cold shock (Kaplan et al., 2004). We hypothesize that regulatory mechanisms under cold stress induced a concerted change in metabolism that allows the cell to cope with the *adh1* mutant, leading to more dependent metabolic profiles in cold shock or cold stress with cold acclimation, so that we can uncover the functional mechanism of *ADH1* anti-freezing.

In this study, the experimental results suggested a significant response from *ADH1* to cold temperature stress. The overall metabolite profiling of *adh1* and WT to cold stress was analyzed with and without prior cold acclimation. Two hundred sixty-three variables were monitored and 78 identified metabolites were annotated for different types of primary metabolism pathways, including organic acids, carbohydrates, amino acids, amines, phosphates, and lipids. Variations in the accumulation of metabolites were detected in the *adh1* and WT plants including soluble sugars and amino acids during both the freezing treatment and the recovery stage. Of particular interest was that the *adh1* mutant caused accumulation of low levels of sucrose, glycine, fumaric acid, glutaric acid, succinic acid, and maleic acid in plants during the freeze treatment in comparison to the optimum temperature. In addition, a correlation-based network analysis revealed that *adh1* effected the normal connectedness of some metabolites (e.g., melibiose, fumaric acid, succinic acid, glycolic acid, and xylose) when the plants were exposed to the cold shock treatment. The findings are discussed to further understand the function of *ADH1* in the response to cold stress in metabolomics and expose the relationship between *ADH1* and cold response in plants.

## Materials and methods

### Plant materials and growth conditions

*Arabidopsis thaliana* (ecotype Columbia), wild type (WT), and *adh1* mutant seedlings were cultured in a growth chamber (16 h light and 8 h darkness at 23°C) after a 2–4 day vernalization period. For growth under sterile conditions, seeds were surface-sterilized (15 min in 20% bleach, 10 min in 70% ethanol, and rinsed thrice with sterile distilled water) and sown on half-strength Murashige and Skoog (MS) medium supplemented with 1% sucrose and 0.8% agar (pH of 5.7), as previously described (Song et al., 2010). Seedlings were cultivated in Petri dishes for 5 days, were subsequently transferred into potting soil and cultured in a growth house at 23°C (16 h light and 8 h darkness) for 3 weeks. Non-acclimated plants were kept in this optimal environment, while cold acclimation was achieved by subjecting 3-week-old plants to a temperature of 4°C for 7 days.

### Chilling and freezing treatments

Chilling and freezing treatments were conducted in low-temp incubators (SANYO, MIR-254, Japan). For chilling stress, 3-week-old WT seedlings were subjected to 4°C for 0, 0.5, 3, 6, 12, 24, 48, and 72 h (for the *ADH* expression assay). Three plants were randomly selected and used for RNA extraction at indicated time points. For the freezing treatment, 3-week-old WT plants and *adh1* mutants with both cold acclimation and non-acclimation were simultaneously treated in a −6°C incubator for 2 h, then all plants were transferred into the growth house at 23°C (16 h light and 8 h darkness) for a 24 h recovery period. Control plants were kept in the growth house and sampled with treatments at the indicated time.

All seedlings were rapidly harvested, flash-frozen in liquid nitrogen, and stored at −80°C until RNA and metabolite extraction.

### Semi-quantitative RT-PCR and real-time qPCR analysis

Total RNA was extracted using the RNA purification kit (Tiangen, Beijing, China) following the manufacturer's instructions. A total of 2 μg of RNA was used for the first-strand cDNA synthesis using reverse transcriptase (Thermo Scientific, Germany) after incubation at 42°C for 1 h 20 min. Semi-quantitative RT-PCR was performed using *CbADH1* and *CbADH2* specific primers. PCR was performed with 30 cycles for the expression of *ADH* assays in the mutant seedlings. The reference gene *EF4A* (TAIR: AT5G60390) was amplified under identical conditions as loading control. The PCR products were separated on 1% agarose gels and stained with ethidium bromide. The utilized primer pairs were *ADH1* (F: 5′-ATGTCTACCACCGGACAGATT -3′ and R: 5′- CATGGTGATGATGCAACGAA -3′), and *EF4a* (F: 5′-TTGGCGGCACCCTTAGCTGGATCA-3′ and R: 5′-ATGCCCCAGGACATCGTGATTTCAT -3′). The real-time qPCR analysis was performed with Platinum SYBR green I qPCR reagents (Takara Bio Inc., Tokyo, Japan). Three technical repeats per sample were used to perform qPCR analysis. UBQ5 (TAIR: AT3G62250) was used as a reference gene to normalize the expression level. The PCR conditions were 35 s at 95°C followed by 40 cycles of the following: 10 s at 95°C, 15 s at 56°C, and 20 s at 72°C. The utilized primer pairs were *ADH1* (F: 5′-TATTCGATGCAAAGCTGCTGTG-3′ and R: 5′- CGAACTTCGTGTTTCTGCGGT-3′) for the expression of *ADH1* assay in mutant seedlings (Licausi et al., 2010), *ADH1* (F: 5′-GTCTTGGTGCTGTTGGTTTAGGC-3′ and R: 5′- GCTTGTCATGGTCTTTCGGGTT-3′) for the expression of *ADH1* assay under low-temperature stress, *ADH2* (F: 5′-CTTTGAGTGCATCGGGAATGTCT -3′ and R: 5′-GGGTTCGACTCTTGAAACCACCA -3′), and *UBQ5* (F: 5′-AAGGTTCAGCGTTTGAGGAAGG -3′ and R: 5′-TCTTTCTGGTAAACGTAGGTGAGTC -3′) (Table S1).

### Electrolyte leakage tests

Electrolyte leakage tests were performed as previously described (Rohde et al., 2004). The third to fifth leaves from 18-day old plants were added to capped test tubes (15 ml) and placed in a bath-type programmable freezer (XT5201-D31-R40C, XuTemp, China). The petiole was placed facing downward into the bottom of the tube (without damaging the leaves). Water was added to the bottom of the tube until the petiole was submerged. Samples were kept on ice for 30 min. The plants were exposed to freezing temperatures ranging from 0 to −20°C (0, −4, −6, −8, −10, −12, −14, −16, −18, and −20°C). Tubes were placed on ice after removal from the bath until removal of all tubes was completed. Leaves were immersed in 10 ml distilled water and placed on a shaker for 2 h at 4°C. Electrolyte leakage was determined as the ratio of before and after sample boiling via a conductivity meter (DDSJ-308, Leici, China). The freezing temperature causing a 50% electrolyte leakage (TEL50) was calculated from plotted data of relative electrolyte leakage as the log EC50 value of sigmoidal curve fitting to the leakage values using SPSS PASW Statistics 18.0 (IBM SPSS software, USA).

### Metabolic profiling analysis

Stress responses of plants were examined during the short-term cold stress of −6°C for 2 h, subsequently removing the stress and analyzing the recovery response over the following 24 h at 23°C, with cold acclimation (4°C 7 d) and de-acclimation, respectively.

The sampling, extraction, storage, derivatization, standard addition, sample injection and metabolite identification/annotation were performed as previously described (Lisec et al., 2006; Fernie et al., 2011). All shoots samples were collected and froze immediately with liquid nitrogen and kept at −80°C until further analysis. Samples (about 100 mg of fresh weight) stored at −80°C were ground using liquid nitrogen and transferred into pre-chilled 2 ml centrifuge tubes. One thousand two hundred microliters of 100% methanol (pre-cooled at −20°C) was then added and vortexed for 10 s, followed by adding 60 μl of ribitol (Sigma, 0.2 mg/ml stock in dH2O) as an internal quantitative standard and vortexed for 10 s. The tubes were placed into an ultrasound machine at 70°C for 30 min, and then centrifuged for 10 min at 11,000 g and 1 ml supernatant was transferred into 10 ml glass centrifuge tubes. After adding 750 ul chloroform (pre-cooled at −20°C) and 1500 μl deionized water (dH2O) (4°C), the tubes were vortexed for 30 s, then centrifuged for 15 min at 3000 g. One thousand microliters supernatant was transferred into a new eppendorf tube. Samples were blow-dried by moderate nitrogen. Fifty microliters of 15 mg/ml methoxyamine pyridine solution was then added, vortexed for 30 s and reacted for 90 min at room temperature. Finally, 50 μl BSTFA reagent (containing 1% TMCS) was added into the mixture, reacted for 60 min at 70°C. After the above reactions, samples were determined for contents of metabolites using Agilent 7890A GC system coupled to an Agilent 5975C inert XL EI/CI mass spectrometric detector (MSD) system (Agilent Technologies, Santa Clara, CA, USA). Furthermore, a mixed n-alkane standard solutions C8–C20 and C21–C40 (Sigma-Aldrich) was used for the determination of Retention indices (RI).

For GC-MS analysis, Gas chromatography was performed on a HP-5MS capillary column (5% phenyl/95% methylpolysiloxane 30 m × 250 μm i.d., 0.25 μm film thickness, Agilent J & W Scientific, Folsom, CA, USA) to separate the derivatives at a constant flow of 1 mL/min helium. One microliter of sample was injected in split mode in a 20:1 split ratio by the auto-sampler. Injection temperature was 280°C, the interface set to 150°C and the ion source adjusted to 230°C. The programs of temperature-rise was followed by initial temperature of 80°C for 5 min, 20°C /min rate up to 300°C and staying at 300°C for 6 min. Mass spectrometry was determined by full-scan method with range from 35 to 500 (m/z).

For quality control (QC) samples, aliquots (about 40 μl) of all prepared sample extracts were mixed as previously described (Sangster et al., 2006). Furthermore, empty vials referred to as “blank controls” were included into the measurement sequence to test for laboratory contaminations.

### Bioinformatics analysis

For metabolite annotation process, raw GC/MS data was converted into CDF format (Net CDF) files via Agilent GC/MS 5975 Data Analysis software (MSD ChemStation workstation) and subsequently processed peaks identification, peaks filtration and peaks alignment via XCMS (www.bioconductor.org). XCMS's default set with the following changes: xcmsSet (fwhm = 3, snthresh = 3, mzdiff = 0.5, step = 0.1, steps = 2, max = 300), group (bw = 2, minfrac = 0.3, max = 300). The signal integration area of each metabolite was normalized to the internal standard (ribitol) for each sample. Identification of metabolites using the Automatic Mass Spectral Deconvolution and Identification System (AMIDS) was searched against commercial available databases such as National Institute of Standards and Technology (NIST) and Wiley libraries. Metabolites were confirmed via comparison of mass spectra and retention indices to the spectra library using a cut-off value of 70% (Abu Dawud et al., 2012).

For multivariate statistical analysis, the XCMS output was further processed using Microsoft Excel (Microsoft, Redmond, WA, USA) and the normalized data was imported into the Simca-P software version 11.0 (Umetrics AB, Umea, Sweden, www.umetrics.com/simca) (Eriksson et al., 2006) for multivariate statistical analyses, including PCA and PLS–DA. All data were mean-centered and unit variance (UV)-scaled before PCA and PLS–DA applied in order to guard against overfitting. A default 7-fold (Leave-1/7th samples-Out) cross validation procedure and 100 random permutations testing were carried out to guard against over-fitting of supervised PLS-DA models. These discriminating metabolites were obtained using a statistically significant threshold of variable influence on projection (VIP > 1.0). Values were obtained from the PLS–DA model and were further validated via student's *t*-test (*P* < 0.05). The metabolites with VIP values above 1.0 and *P* values below 0.05 (threshold) were selected as discriminating metabolites between two classes of samples.

Hierarchical cluster analysis (HCA) was performed and visualized using the pheat map package available in R 3.0.3 version (www.r-project.org). The data were centered by the average of the controls to create heatmap. Pearson's product-moment correlation (Pearson's ρ) coefficient of 0.9 was performed to calculate the correlation. Corresponding *P*-values and FDR (false discovery rates) (Benjamini et al., 2001) of each correlation were also calculated using the Cor. test function in R.

To identify coordinated cold treatments induced metabolic perturbation, correlation-based network was used (Hochberg et al., 2013). Pearson's product–moment correlation (Pearson's ρ) was performed on each of the six matrices of data profiles obtained from the two genotypes (WT and *adh1*) under the three conditions [the control group (CK), cold shock (S), and cold acclimation (A)]. *P* value was corrected with false discovery rate (FDR). Correlation matrix was calculated using cor. and cor. test function available in R 3.0.3 version (www.r-project.org). The adjacency matrices were construct via the significant correlation (|r| > 0.9 and FDR < 0.05). Network communities and properties were computed by the igraph R package V0.7.1 (http://www.igraph.org/r/). Average node degree or average links: the average number of edges per node in the network; network density: characterizing the proportion of edges in a network in relation to the total number of all possible edges in the network (Barabási and Oltvai, 2004; Hochberg et al., 2013). The above properties were investigated. For comparative analysis, the network intersections and network symmetric difference were determined among the optimum temperature, cold shock and cold acclimation, between *adh1* and WT. Between-genotype differences are visualized in the symmetric difference networks (SDN) by R. Cytoscape (Shannon et al., 2003) version 3.2.0 (http://www.cytoscape.org/).

Identified metabolites were mapped onto general biochemical pathways according to the annotation in KEGG (Kyoto Encyclopedia of Genes and Genomes) (Kanehisa and Goto, 2000) (http://www.genome.jp/kegg/). Metabolic pathway maps were constructed by incorporating the identified and annotated metabolites using R 3.0.3 version (www.r-project.org) and Power Point (Microsoft, Redmond, WA, USA).

## Results

### Low temperature induced *ADH1*

**Figure 1:**
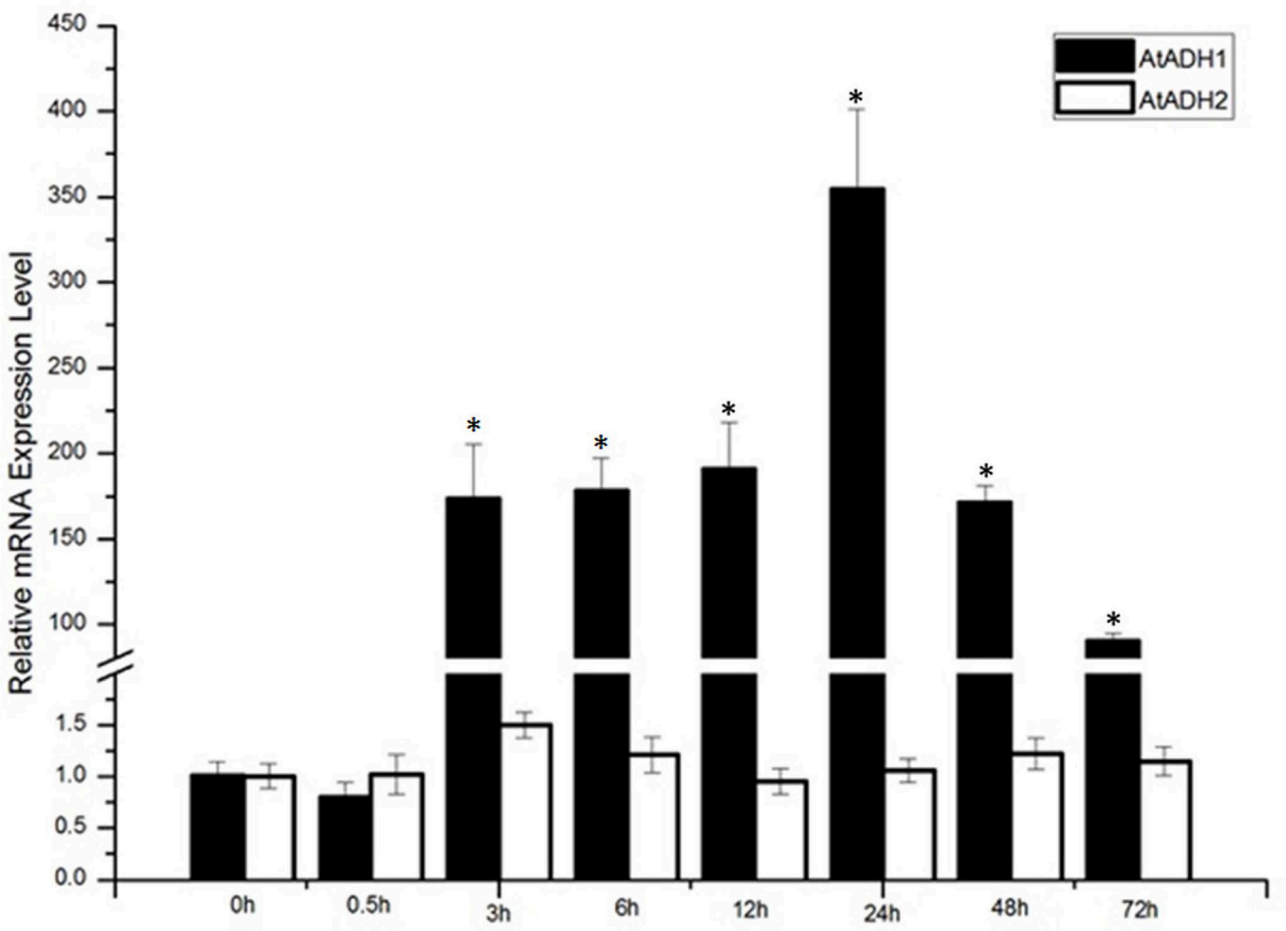
**The expression levels of ***AtADH1*** in response to −4°C cold stress treatments**, *CbADH1* gene expression induced by cold stress. Conditions for Q-PCR were optimized to show induction relative to basal levels (time 0). The data represented the average of triplicate experiments ± standard deviation (SD). The asterisk indicated significant difference (*p* < 0.001).

In order to verify whether the *ADH* gene was induced by low temperatures, the expression levels of *ADH1* and *ADH2* at 4°C were tested via qPCR analysis. Conditions were optimized to show induction relative to basal levels (time 0 h). Following treatment with 4°C for 72 h, the *ADH1* transcript level significantly increased, whereas *ADH2* showed no significant increase (Figure 1). The 4°C stress caused *ADH1* transcript induction at 3 h following stress treatment, and the expression level declined after initial rise due to 24 h treatment.

### Isolation of *ADH1* knockout mutants

**Figure 2:**
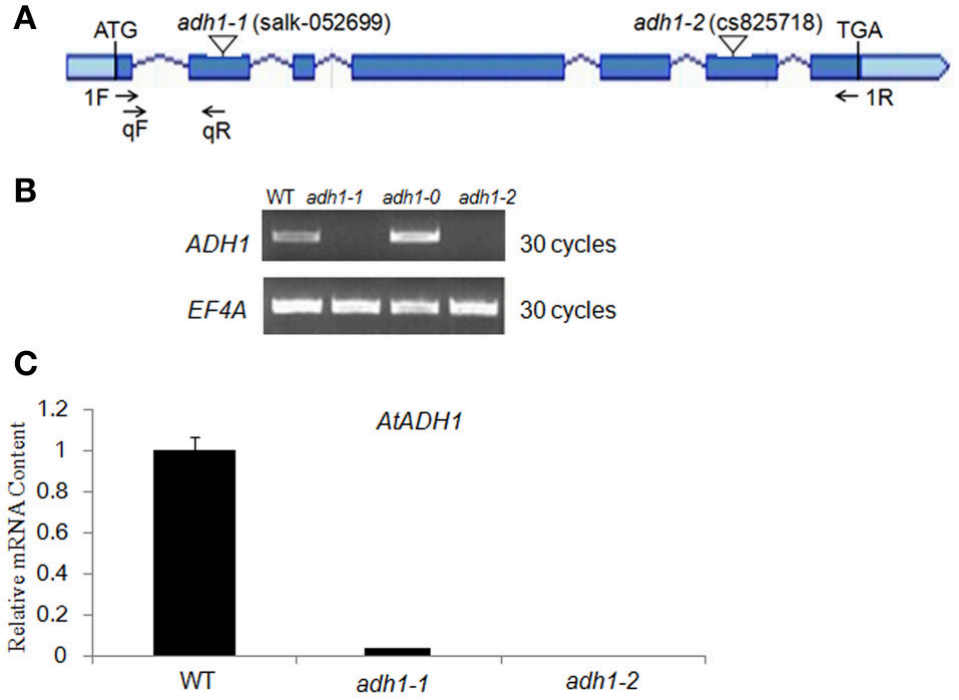
**Characterization of ***adh1*** mutants. (A)** Schematic diagram showing the positions of the T-DNA insertions or nucleotide changes at the *ADH1* locus. Rectangles represent exons. Arrows indicate PCR primers used in genotyping and RT-PCR analysis of T-DNA alleles. **(B)** Genotyping of *adh1* T-DNA alleles. **(C)** RT-PCR analysis of *AtADH1* transcript level in *adh1* T-DNA mutants, EF4A as the reference gene.

To further reveal the *in vivo* role of *ADH1* for plant cold tolerance, we isolated T-DNA mutants with T-DNA insertion in the exon of *ADH1* [*adh1-1* (SALK_052699) and *adh1-2* (SALK_825718)] (Figures 2A–C). Using semi-quantitative RT-PCR analysis, the transcript of *ADH1* was tested in *adh1-1* and *adh1-2* mutants (Figure 2B). Quantitative real-time PCR analysis showed that the transcript level of *ADH1* was completely inhibited in the *adh1-2* mutant (Figure 2C).

### The *adh1* mutant lacks tolerance to freezing damage

To determine the cold hardiness of the *adh1* mutant, the electrolyte leakage rates in WT, *adh1-1*, and *adh1-2* were tested after freezing treatments in the panel, and their semi-lethal temperatures (LT_50_) were calculated based on changes in electrolyte leakage rates under different low-temperature stresses using the logistic equation.

**Figure 3:**
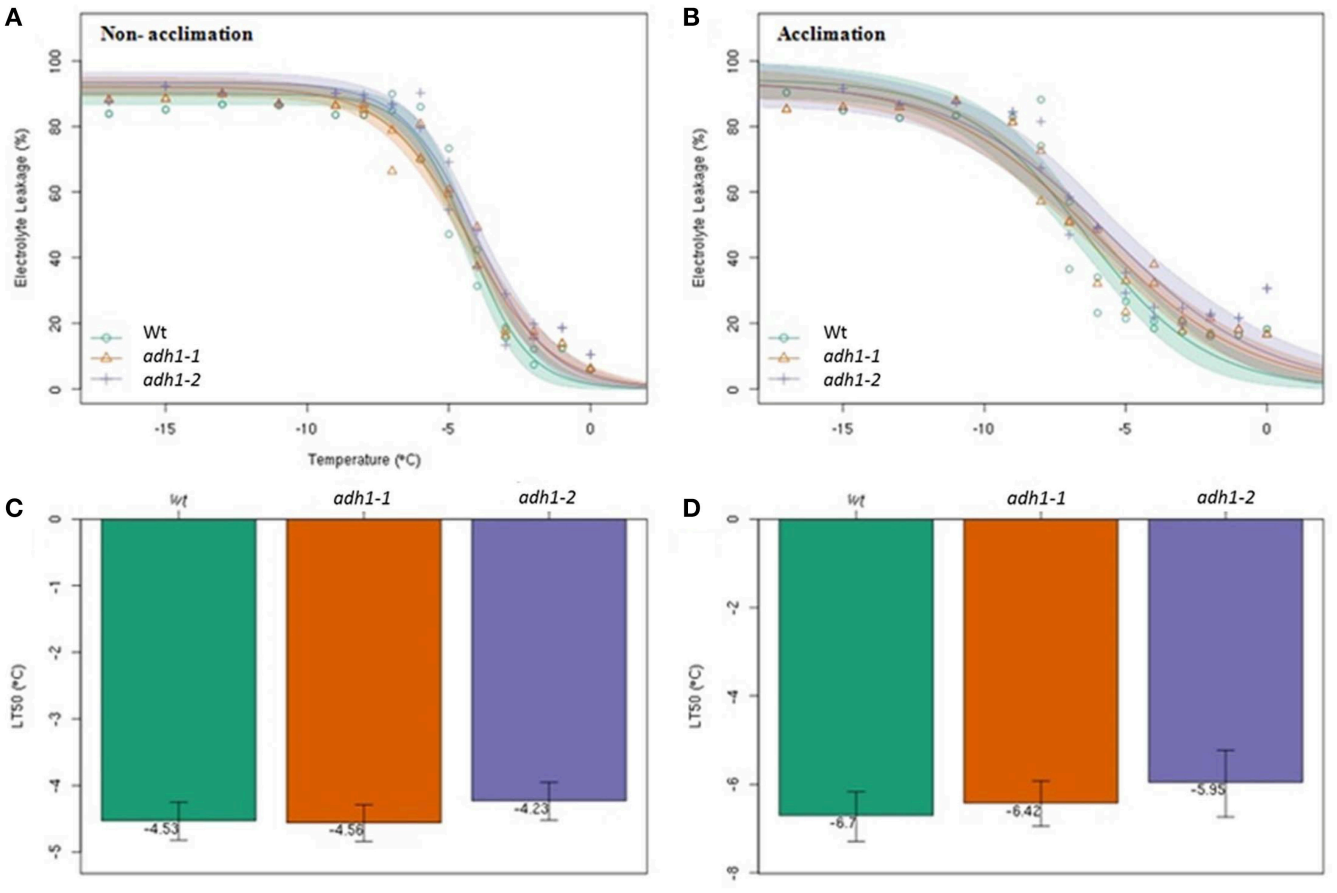
**The ***adh1-2*** mutant is less tolerant to freezing damage**. The semi lethal temperature (LT50) was determined according to the electrical conductivity in Wild-type and *adh1-1* and *adh1-2* mutant plants. The error bars indicate standard errors (*n* = 6). **(A,C)** All plants were grown at 23°C for 5 weeks, and direct determined of the conductivity and the semi lethal temperature. **(B,D)** All plants were grown at 23°C for 4 weeks, and 4°C for 7 d, following determination of the conductivity and the semi lethal temperature.

The results revealed that with decreased temperature and prolonged treatment time, the electrolyte leakage rates showed an S-shaped curve (Figure 3). The semi-lethal temperatures (LT_50_) of WT, *adh1-1* (SALK_052699), and *adh1-2* (SALK_825718) were not significantly different (Figures 3A,C). However, electrolyte leakage rates of WT, *adh1-1*, and *adh1-2* subsequent to cold-acclimation (4°C for 7 d) were −6.7°, −6.42°, and −5.95°C, respectively (Figures 3B,D). The *adh1-2* mutant showed a higher percentage of ion leakage than WT after freezing treatment, suggesting that plasma membranes were more damaged in the mutant. Without cold adaptation, *adh1-2* also showed lower basal freezing tolerance than WT plants.

### Metabolome profiling of *adh1* mutant treated freezing stress

**Figure 4:**
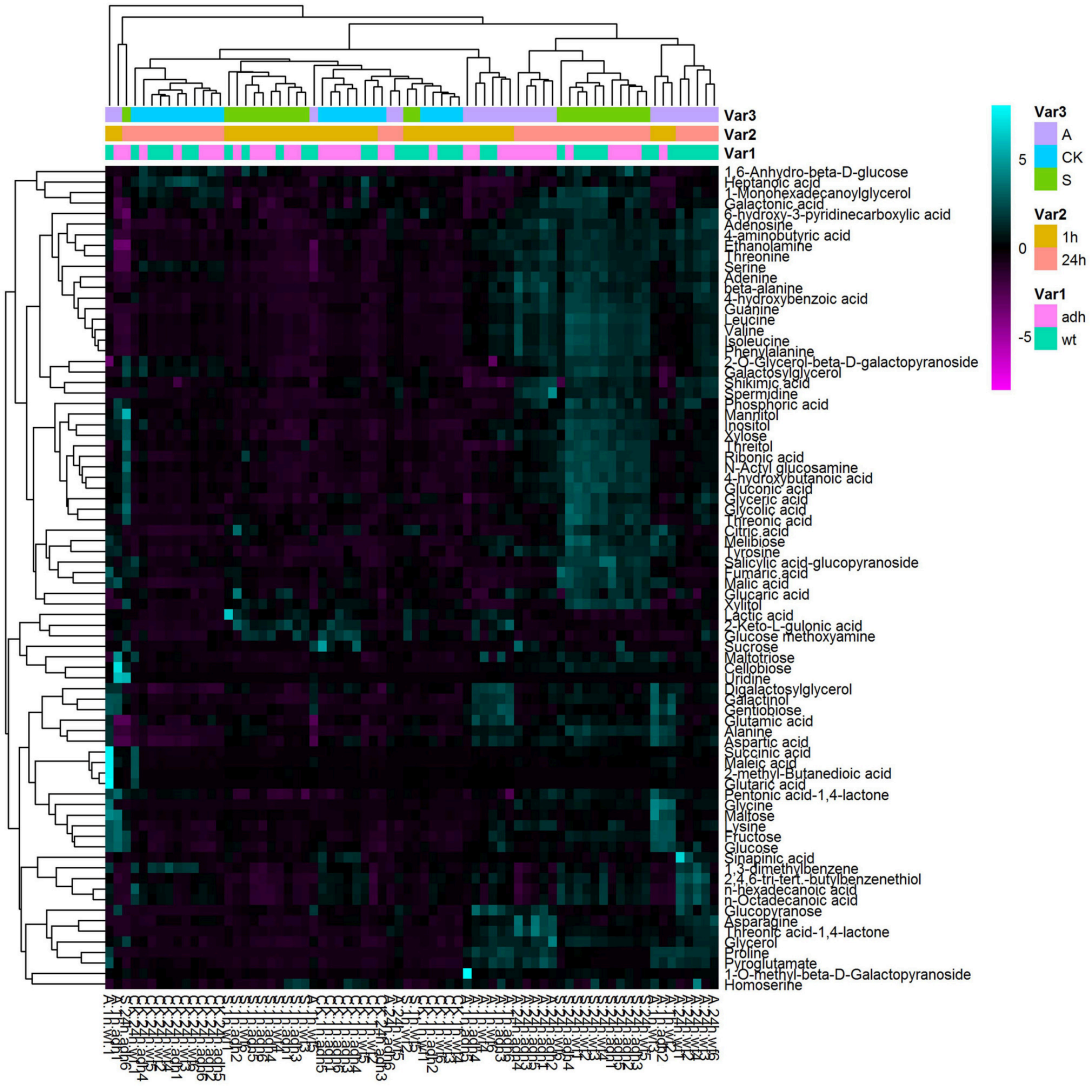
**Heatmap analysis of the metabolite profile**.

To better evaluate the role of *ADH1* in plant freezing tolerance, the metabolites profiling *adh1-2* responses were analyzed via GC-MS. Treatments were −6°C for 2 h with non-acclimation and cold acclimation (cold shock and cold acclimation) followed by growth at 23°C for 24 h (recovery). In total, 263 resolved peaks were identified, among which 78 could be identified as discrete metabolites with known chemical structure (Supplementary File 1). A considerable amount of material has not been annotated as a result of low signal or missing database. The results revealed that amino acids, organic acids, and several other substances changed in the treatment groups (Figure 4).

**Figure 5:**
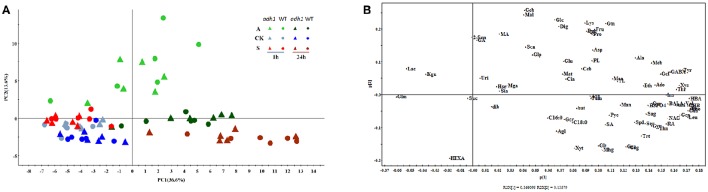
**Principal component analysis (PCA) of metabolic profiles in ***adh1*** mutant response to cold stress (six biological replicates)**. All samples of this investigation are represented. **(A)** The score plot of PCA model. A, Freezing treatment with cold acclimation (light green) and recovery at 23°C 24 h (deep green); CK, control samples living in 23°C always (blue); S, freezing shock without cold acclimation (light red) and recovery at 23°C 24 h (deep red), *adh1* mutant (triangles), and wild type (circles) are shown. Time points are indicated within the graph. **(B)** The loading plot for the PCA model.

Principal component analysis (PCA) of all samples was performed (Figure 5), revealing that the metabolite differences were not significant between WT and *adh1-2*. However, they were significantly different between cold shock, cold acclimation, and room temperature controls. The time series effects (freezing treatment for 2 h and recovery for 24 h in optimal temperature) clearly contributed most to total variance within the dataset (Figure 5).

In the GC/MS-based analysis of central metabolites, the first principal component (PC1) and the second principal component (PC2) explain the highest amount of variance (50.2%) across the dataset, separating the samples (Figure 5B). PC1 explained 36.6%, and Phenylalanine, Isoleucine, and 4-hydroxybenzoic acid were the main metabolites contributing to the dispersion of samples on PC1 (Figure 5B). The second component (PC2) explained 13.6% of the variance and separated the samples (Figure 4A). Changes in the abundance of glucose, maltose, and gentiobiose were predominantly responsible for sample dispersion along PC2 (Figure 5B). Detailed information is presented in the supplementary file (Supplementary File 2). The PCA results are consistent with PLS–DA analysis (Figure S1).

### Metabolic signatures of the *adh1* mutant treated to freezing stress

**Figure 6:**
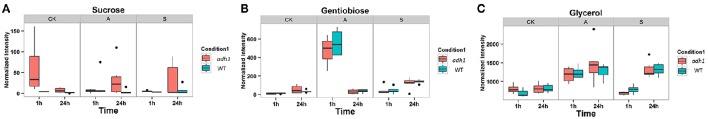
**Boxplot-visualizations of significantly changed metabolites relative abundances in control and cold treatments. (A)** Sucrose responsed on different treatments. **(B)** Gentiobiose responsed on different treatments. **(C)** Glycerol responsed on different treatments.

To better evaluate the changes in the level and metabolism of each metabolite, students' *T*-test was used to reveal that the levels of 78 metabolites significantly varied (*p* < 0.05). These included several soluble sugars, amino acids, organic acids, nucleotides, phosphates, and glycerol, as well as sugars such as sucrose, glucopyranose, melibiose, gentiobiose, maltose, fructose, cellobiose, xylose, inositol, maltotriose, and glucose (Table 1), suggesting that *ADH1* affected the accumulation of soluble sugars as cold stress response. For example, sucrose (*P* < 0.05, fold-change = −14.723 in the control group at 23°C) was significantly reduced in the background level in WT compared to *adh1* seedlings. However, when cold acclimation preceded freezing stress, this gap of sucrose content had narrowed, and sucrose was significantly increased in WT compared to *adh1* mutants of seedlings (*P* = 0.382, fold-change = 2.751 in the freezing treatment −6°C for 2 h in the cold acclimation group; *P* = 0.446, fold-change = 1.959 in recovery at 23°C for 24 h). Of particular interest were the cold shock results, where the reduced state was maintained continuously (*P* < 0.05, fold-change = −1.558 in the cold shock group −6°C for 2 h; *P* = 0.241, fold-change = −4.141 in recovery at 23°C for 24 h) (Table 1 and Figure 6A). A similar situation was detected for glucopyranose and xylitol. However, gentiobiose increased in the background level in WT compared to *adh1* seedlings but significantly decreased in the freezing treatment with cold acclimation of 24 h, and changed only a marginally in the cold shock treatment (Table 1 and Figure 6B). Glycerol showed an increase in cold treatments but remained basically the same between WT and *adh1* in the control group at 23°C (Table 1 and Figure 6C). Detailed information is presented in Table 1 as well as in Supplementary File 1.

**Table 1:** **Metabolic responses to freezing stress in wild type and ***adh1*** mutant**.

**Compare**	**WT vs**. ***adh1***						**A vs. CK**				**S vs. CK**			
**Group**	**CK**		**A**		**S**		***adh1***		**WT**		***adh1***		**WT**	
**Time**	**1 h**	**24 h**	**1 h**	**24 h**	**1 h**	**24 h**	**1 h**	**24 h**	**1 h**	**24 h**	**1 h**	**24 h**	**1 h**	**24 h**
Glucopyranose	−**1.83**	−**1.79**	−0.36	0.02	−0.42	0.76	**1.75**	**4.19**	**3.21**	**6.16**	−0.20	**2.05**	1.21	**4.60**
Melibiose	0.45	−0.66	0.42	−**1.55**	0.36	0.36	**3.93**	0.61	**3.90**	**1.12**	**2.39**	**2.07**	**2.29**	**3.09**
Guanine	−**0.64**	−0.76	−0.37	**1.45**	0.33	0.33	0.50	**1.71**	0.77	**1.95**	−**1.00**	**1.57**	−0.04	**2.67**
Citric acid	−0.68	−0.63	0.04	−0.30	−0.08	−0.01	−0.15	**0.98**	0.58	**1.58**	0.41	**2.00**	**1.02**	**2.62**
Proline	−0.31	−0.51	−0.13	−0.16	0.31	0.50	**4.14**	**3.37**	**4.31**	**4.08**	−0.03	**1.60**	**0.58**	**2.61**
Malic acid	−0.44	−0.34	0.01	−0.40	0.06	0.00	−0.10	**0.81**	0.36	**1.14**	−0.54	**2.11**	−0.03	**2.45**
Sucrose	−**3.88**	−**2.38**	1.46	0.97	−**0.64**	−2.05	−3.27	2.19	2.06	1.47	−**3.48**	2.03	−0.24	2.35
Spermidine	−0.42	−**1.41**	−0.55	**3.53**	0.24	0.12	−**1.27**	**1.59**	−1.39	**2.50**	−**1.70**	0.78	−1.04	**2.31**
Threonic acid-1,4-lactone	−0.16	−**0.48**	0.01	**1.47**	0.47	0.59	**1.76**	**3.05**	**1.92**	**2.68**	0.00	**1.16**	0.64	**2.22**
Gluconic acid	0.16	−0.44	0.05	**0.58**	0.19	0.13	**1.22**	**0.73**	**1.11**	**1.07**	**0.87**	**1.63**	0.90	**2.20**
Glycine	−0.01	−0.20	1.13	−0.95	0.32	0.23	1.70	**2.12**	2.84	**1.96**	−**0.73**	**1.72**	−0.40	**2.15**
Alanine	−0.25	−**0.32**	0.34	−0.44	0.11	0.15	**1.11**	**1.28**	**1.70**	**1.33**	**0.88**	**1.63**	**1.24**	**2.11**
Gentiobiose	0.28	−0.70	0.23	−**4.20**	0.04	0.21	**5.41**	−0.83	**5.36**	0.16	1.94	1.16	1.70	**2.07**
Phenylalanine	−**0.42**	−0.33	−0.17	**1.09**	0.12	0.34	0.80	**1.40**	1.05	**1.31**	−0.18	**1.38**	0.36	**2.06**
Isoleucine	−0.27	−0.16	−0.11	**0.89**	0.23	0.39	**1.06**	**1.34**	**1.22**	**1.13**	−0.09	**1.47**	**0.41**	**2.02**
Tyrosine	−0.38	−0.34	0.18	0.09	**0.74**	0.16	**1.03**	**1.23**	**1.59**	**0.98**	−0.46	**1.43**	0.66	**1.92**
Fumaric acid	−0.55	0.57	1.00	−1.27	0.24	0.09	−0.76	−0.20	0.79	−1.23	0.05	**2.41**	**0.84**	**1.92**
Aspartic acid	−0.35	−**0.65**	0.22	−0.17	0.14	**0.70**	0.08	**1.10**	0.65	**1.71**	−**0.43**	0.50	0.07	**1.85**
Maltose	−0.23	−0.04	0.76	−1.84	0.32	−0.11	2.04	**0.83**	**3.03**	0.92	**0.59**	**0.78**	**1.15**	**0.71**
Leucine	−0.32	−0.16	−0.25	**1.09**	0.14	0.35	0.66	**1.09**	0.73	**0.82**	−0.07	**1.29**	0.39	**1.81**
beta-alanine	−0.11	−0.07	0.10	**1.27**	0.24	0.50	1.00	**1.88**	**1.21**	**1.62**	−**0.41**	**1.20**	−0.06	**1.77**
Valine	−0.23	−0.13	−0.13	0.66	0.16	0.38	**1.10**	**1.16**	**1.21**	**0.98**	0.19	**1.21**	**0.59**	**1.72**
Glycolic acid	−**0.40**	0.16	−0.07	−0.29	0.08	−0.10	0.26	**0.53**	**0.59**	**0.90**	−0.15	**1.90**	0.33	**1.64**
Threonic acid	−**0.55**	−0.24	−0.13	**0.62**	−0.08	0.07	0.02	**0.35**	**0.44**	**0.64**	0.22	**1.28**	**0.69**	**1.59**
Xylitol	−**0.31**	−0.03	0.27	**0.97**	−0.10	−0.04	−**1.88**	0.24	−**1.30**	0.19	−0.01	**1.57**	0.20	**1.57**
4-aminobutyric acid	−0.40	0.14	0.11	0.28	0.48	0.30	**1.24**	**1.61**	**1.74**	**1.57**	−0.20	**1.30**	**0.67**	**1.46**
Adenosine	−0.19	−0.09	0.25	**0.55**	0.11	0.32	0.00	**1.53**	0.43	**1.59**	−**0.73**	**1.01**	−0.44	**1.42**
Ribonic acid	−0.21	−0.33	−0.04	**0.46**	0.17	0.04	0.03	**0.36**	0.21	**0.64**	0.03	**1.01**	0.42	**1.38**
4-hydroxybutanoic acid	−0.14	−0.04	0.11	**0.37**	0.29	−0.04	**0.59**	**0.77**	**0.84**	**0.52**	−0.04	**1.35**	**0.40**	**1.35**
Phosphoric acid	**0.85**	−0.08	−1.15	**1.54**	0.35	−0.13	1.72	**0.93**	−0.28	**1.06**	0.27	**1.36**	−0.24	**1.31**
Fructose	−0.36	−0.18	0.24	−**1.03**	0.07	−0.19	**1.26**	**0.55**	**1.86**	**0.57**	0.21	**1.29**	**0.64**	**1.29**
Cellobiose	−0.25	−0.07	−0.87	0.28	0.35	−0.17	1.67	**0.88**	**1.06**	**0.72**	−**0.42**	**1.33**	0.18	**1.23**
Threonine	−0.15	−0.15	0.14	0.55	0.22	0.36	0.35	**1.06**	0.64	**1.15**	−**0.28**	0.70	0.10	**1.21**
Threitol	0.04	0.00	0.31	**0.94**	0.37	−0.03	−0.44	**0.50**	−0.17	**0.36**	0.18	**1.22**	0.51	**1.19**
Adenine	−0.30	−0.36	−0.31	**1.04**	0.21	0.29	**0.82**	**0.86**	0.82	**0.75**	−0.24	0.49	0.28	**1.14**
4-hydroxybenzoic acid	−0.10	0.23	−0.25	**0.72**	0.10	0.16	**0.90**	**1.22**	**0.75**	**0.78**	−0.17	**1.19**	0.03	**1.13**
Mannitol	−0.18	−0.13	0.01	0.27	0.17	−0.37	0.35	0.34	**0.54**	**0.51**	−**0.27**	**1.34**	0.08	**1.10**
N-Actyl glucosamine	−0.12	−0.04	0.26	0.26	0.17	−0.08	0.08	**0.33**	**0.46**	0.19	−0.23	**1.14**	0.06	**1.09**
2-Keto-L-gulonic acid	−**2.53**	0.28	−0.95	−**0.86**	−0.30	−0.25	−0.16	**1.01**	**1.42**	**1.46**	0.13	**1.55**	**2.36**	**1.01**
Asparagine	−0.81	−0.61	−0.31	**1.95**	0.45	−0.37	0.23	**1.84**	0.74	**2.03**	−**1.43**	0.33	−0.17	0.57
Salicylic acid-glucopyranoside	−0.45	0.22	0.22	−0.10	0.23	0.09	0.16	−0.03	**0.83**	−0.08	−0.05	**1.12**	**0.63**	**0.99**
Glyceric acid	−**0.29**	−0.04	0.05	**0.63**	−0.02	0.11	−**0.63**	**0.46**	−0.29	**0.69**	−**0.68**	**0.79**	−**0.42**	**0.94**
Xylose	−0.04	0.04	0.05	0.09	0.26	−0.07	**0.72**	**0.58**	**0.81**	0.19	−0.19	**0.93**	0.12	**0.82**
Glucaric acid	−**0.42**	0.08	−1.33	**1.72**	−0.35	−0.29	−0.01	**0.80**	−0.92	0.46	0.49	**1.19**	0.57	0.81
Inositol	−0.18	−0.15	0.07	−0.18	0.11	0.04	**0.54**	−0.02	**0.80**	0.12	−0.06	**0.62**	**0.24**	**0.81**
Shikimic acid	−0.18	0.13	0.68	**1.44**	−0.01	−0.19	−**1.50**	**1.28**	−**0.64**	**1.11**	−0.16	**1.09**	0.01	0.77
Glutamic acid	−0.21	−0.34	0.00	−0.57	0.02	0.49	0.52	−0.12	0.73	0.36	0.02	−0.11	0.24	**0.73**
Galactinol	−0.12	−0.05	0.16	−**0.83**	0.14	0.07	**1.07**	**0.33**	**1.35**	**0.53**	−0.17	**0.59**	0.10	**0.71**
Glycerol	−0.22	0.00	0.06	0.26	0.20	0.01	**0.57**	**0.86**	**0.85**	**0.65**	−**0.22**	**0.70**	0.20	**0.70**
Ethanolamine	−0.14	−0.05	0.18	0.14	0.16	0.34	0.06	**0.49**	0.38	**0.57**	−**0.22**	0.28	0.08	**0.66**
Lysine	−0.20	0.11	0.20	−0.26	0.20	−0.44	1.04	**1.42**	**1.45**	**0.79**	−0.12	**1.21**	0.28	**0.65**
Digalactosylglycerol	−0.17	0.00	0.09	−**0.49**	0.18	**0.29**	**0.33**	**0.48**	**0.60**	**0.56**	−**0.29**	**0.34**	0.06	**0.63**
6-hydroxy-3-pyridinecarboxylic acid	−0.03	0.05	0.30	**0.38**	0.28	0.46	−**0.51**	**0.52**	−0.18	**0.58**	−**0.57**	0.18	−0.26	**0.59**
1-Monohexadecanoylglycerol	0.13	−0.09	0.40	**0.72**	0.17	**0.38**	−**0.71**	−0.28	−0.44	−0.31	−**0.52**	0.10	−0.48	**0.57**
Homoserine	−0.18	0.65	−0.33	−0.65	−0.35	0.01	0.24	0.50	0.09	0.75	−0.22	1.19	−0.39	0.56
2,4,6-tri-tert.-butylbenzenethiol	−0.39	−0.05	−0.12	−0.10	0.19	**0.39**	−0.24	−0.13	0.02	**0.86**	−**0.88**	0.09	−0.31	**0.53**
Galactonic acid	0.47	−0.05	0.23	**1.05**	0.40	0.41	−0.54	0.20	−**0.78**	0.01	−0.42	0.05	−**0.48**	**0.51**
Glucose methoxyamine	−**0.62**	−**0.54**	−0.41	−0.08	−0.18	0.09	−**0.72**	−0.18	−**0.51**	**0.85**	−0.27	−0.16	0.17	**0.48**
2-O-Glycerol-beta-D-galactopyranoside	−0.16	−0.12	−0.56	**1.04**	0.09	0.00	0.00	0.18	−0.40	0.20	−**0.28**	**0.32**	−0.03	**0.44**
1-O-methyl-beta-D-Galactopyranoside	−0.15	−0.11	−0.98	0.11	0.08	0.03	1.42	0.22	**0.59**	0.17	−**0.18**	0.25	0.05	**0.39**
Serine	−0.20	−0.03	0.03	**0.82**	0.33	0.40	0.04	0.15	0.27	0.28	−**0.54**	−0.04	0.00	**0.38**
Maltotriose	−**0.77**	0.43	−0.62	0.02	0.15	−0.04	0.78	**0.45**	0.94	−0.06	−**0.63**	**0.82**	0.30	0.35
Sinapinic acid	−**1.39**	0.02	−0.12	0.09	**0.50**	0.01	−**0.86**	0.55	0.41	1.98	−**1.44**	0.33	0.45	0.33
Galactosylglycerol	−0.08	0.01	0.08	**0.31**	0.11	0.07	−0.21	−0.08	−0.05	−0.06	−0.10	**0.25**	0.09	**0.30**
Glucose	−**0.23**	0.00	−0.03	−**0.46**	0.04	−0.14	**0.46**	0.25	**0.66**	**0.42**	−0.12	**0.44**	0.15	**0.30**
n-Octadecanoic acid	−**0.37**	0.13	−0.05	−0.08	0.14	**0.32**	−**0.37**	−0.31	−0.05	0.31	−**0.66**	0.08	−0.15	0.27
Pentonic acid-1,4-lactone	0.07	0.15	0.33	−0.11	0.07	**0.20**	0.33	0.31	**0.58**	0.07	−0.22	0.19	−0.23	**0.24**
n-hexadecanoic acid	−**0.35**	0.22	−0.03	−0.24	0.14	**0.34**	−**0.36**	−**0.40**	−0.04	0.16	−**0.64**	0.05	−0.15	0.17
Succinic acid	−0.19	1.02	1.35	−1.24	**0.28**	0.01	0.14	**0.82**	1.68	0.07	−**0.33**	**1.15**	0.14	0.13
Uridine	−**0.41**	0.83	−1.41	−0.68	**0.40**	−1.81	4.60	**0.66**	2.60	−0.73	−**0.34**	3.75	**0.47**	0.12
Pyroglutamate	−0.26	**0.50**	0.41	−0.13	0.38	0.23	**2.30**	**1.87**	**2.96**	**1.05**	−0.06	0.25	**0.57**	−0.02
Lactic acid	−0.09	0.15	0.14	−**0.55**	0.61	−0.16	−0.09	**0.49**	0.14	**0.59**	0.12	0.08	0.82	−0.23
1,3-dimethylbenzene	−**0.32**	**0.64**	−0.02	−0.32	0.10	**0.28**	−0.17	−0.31	0.12	−0.22	−**0.40**	−0.02	0.02	−**0.38**
1,6-Anhydro-beta-D-glucose	0.27	−0.40	−0.29	−0.11	−0.47	0.13	1.58	−**1.61**	1.02	−**1.26**	**2.11**	−0.03	1.37	0.50
Maleic acid	−**0.54**	1.85	1.65	−1.23	0.39	−0.28	0.98	**1.31**	3.17	−0.87	−0.25	**1.65**	**0.69**	−0.48
Heptanoic acid	0.08	0.32	0.32	1.18	−0.24	0.04	−**2.08**	−**0.95**	−**1.84**	−**1.01**	−**0.48**	−0.41	−0.80	−**0.69**
2-methyl-Butanedioic acid	−0.10	2.64	1.95	−3.46	0.29	0.29	**0.69**	**0.79**	2.74	−1.47	0.38	**0.70**	**0.76**	−1.64
Glutaric acid	−0.06	4.15	2.84	−4.91	**0.44**	0.32	**1.23**	**1.22**	4.13	−2.49	**0.52**	**0.72**	**1.02**	−3.10

Index 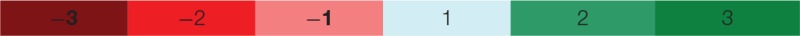

*Values are the logarithmic transformed fold change of selected metabolites on samples. Bolded figures represent significant difference between treatments of samples tested by the Student's t-test (p < 0.05). Different colors represent the increase (green) or decrease (red) in metabolite logarithmic fold change indicated in the color index (n = 6). A, cold acclimation samples treated −6°C 1 h and recovery at 23°C 24 h; CK, control samples living in 23°C always; S, cold shock samples treated −6°C 1 h and recovery at 23°C 24 h*.

Correlation-Based Network Analysis (CNA) to Identify the Freezing Treatment Induced Metabolic Perturbation

**Figure 7:**
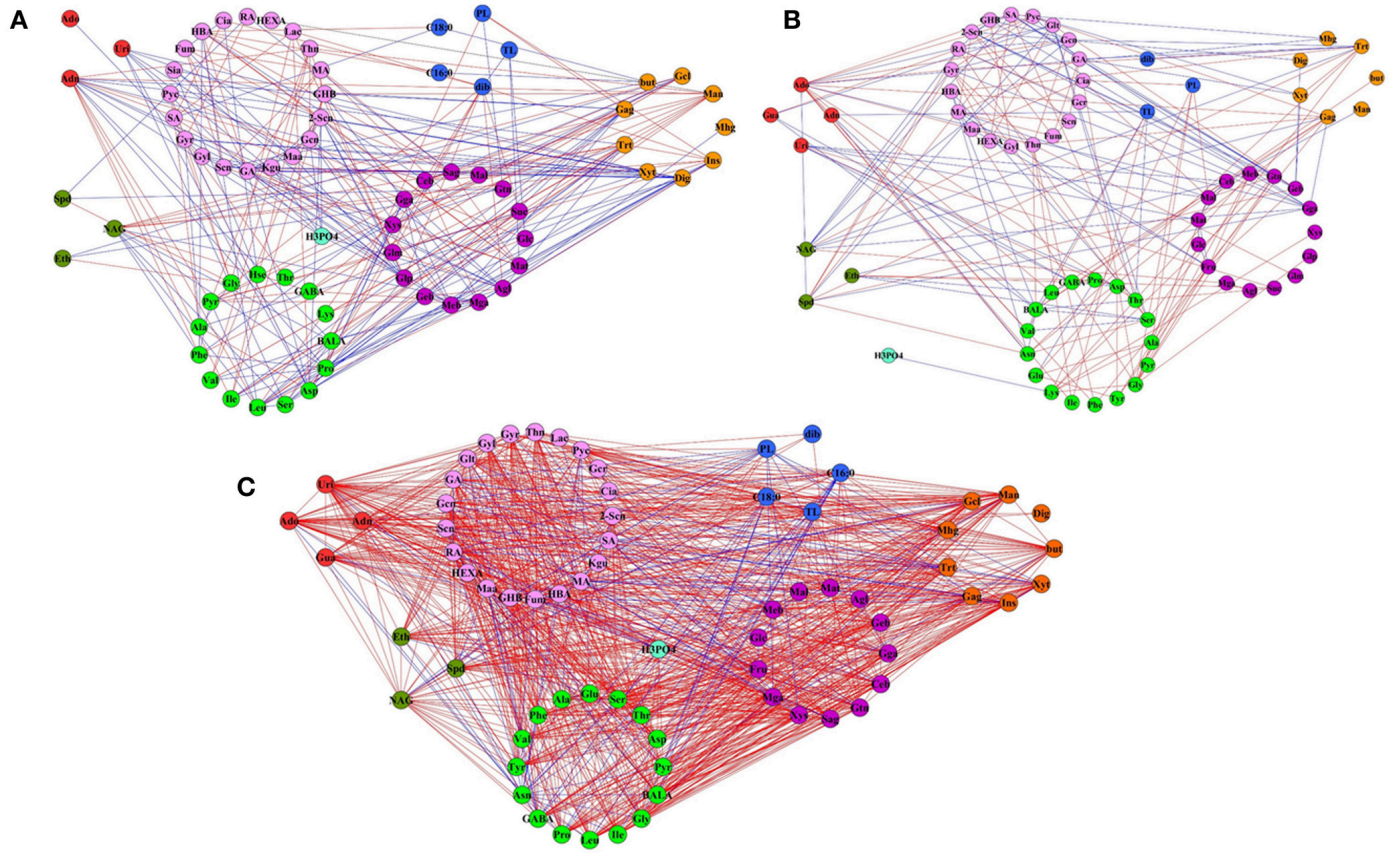
**Metabolite network based on significant correlations in cold treatment samples**. Node colors represent different primary metabolites. Green for the amino acid, lavender for organic acid, purple for sugar, red for the nucleotide, brown for the polyol, dark green for the polyamine, blue for the other. Edges between nodes represent correlations identified as significant at |r| > 0.9, FDR < 0.05, where blue edges specific to *adh1* and red edges specific to WT. **(A)** In the control group (always in 23°C). **(B)** Freezing treatment with cold acclimation (4°C for 7 d, then treated at −6°C for 2 h). **(C)** Freezing shock without cold acclimation (−6°C for 2 h).

To further screen the calculated correlations, seven networks were generated (Supplementary File 3, Figure 7, and Figure S2). At a false discovery rate (FDR) < 0.05 and *r* > 0.8, the network of the *adh1* mutant and WT changed little in both the control group and the cold acclimation group (Figures 7A,B). However, cold shock caused a significant increase in the number of edges in the *adh1* mutant from 334 to 1410, and in WT from 378 to 2700. Network density changed in the *adh1* mutant from 0.105 to 0.309, and in WT from 0.088 to 0.669. The average node degree changed from 11.8 to 42.5, and from 11.5 to 84.4, respectively. In summary, under cold shock, WT networks were characterized by increased numbers of edges compared to *adh1* mutant networks (2700: 1410), which markedly increased by 50% (Figure 7C and Supplementary File 3), suggesting a significant effect of *adh1* mutation on the metabolism under cold shock.

The difference of *adh1* mutant and wild type in metabolic response to cold treatment can be appreciated by comparing the graphs. To further visualize the between-sample effects on metabolic networks, symmetric difference networks (SDN) were utilized (Figure 7), in which blue edges were specific to *adh1* and red edges were specific to WT. The SDN is mainly used for network connections under different conditions and emphasizes the presence of different connected samples specific nodes. E.g., under cold shock, SDN comprised 132 nodes and 1870 edges, 68 nodes were specific to *adh1* and 64 nodes were specific to WT, 290 edges were specific to *adh1*, and 1580 were specific to WT, resulting in a ratio of 1 to 5.45 (Supplementary File 2). Notably, all 40 edges of maleic acid (MA) and all 44 edged of asparagine (Asn) were specific to *adh1*, while 26 edges of maltotriose (Mat) and 42 edges of gentiobiose (Geb) were specific to WT (Supplementary File 3: (S)WT-ADH).

This result revealed the differences of extensive topological networks between *adh1* and WT due to different cold treatments. E.g., in both strains (*adh1* and WT) adenosine played a central role in the structure of the network under cold treatment (cold shock and cold acclimation), but under control conditions (constant mild temperature), most of the connectivity was lost (Supplementary File 3, Figure 7, and Figure S2). Metabolites in *adh1* altered between cold shock (−6°C for 2 h) and cold acclimation (4°C for 7 d, then treated at −6°C for 2 h). According to the connectedness of correlation-based network analysis, melibiose, fumaric acid, succinic acid, glycolic acid, and xylose lost the relations with the network of cold acclimation, but enhanced relations with the network of cold shock. These results have not been found for the WT network.

### Changes in metabolite abundance under cold treatment

**Figure 8:**
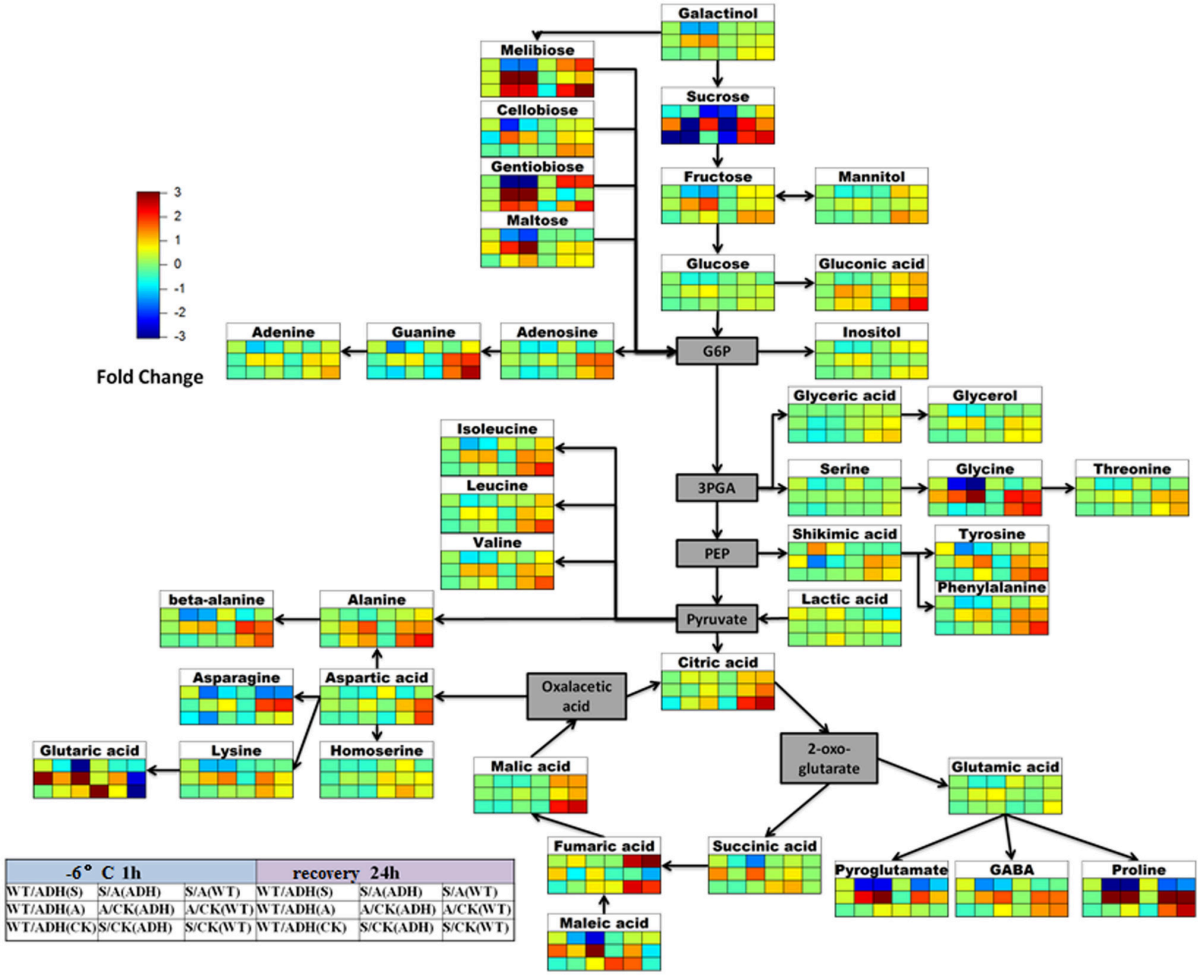
**Scheme of metabolite abundance during cold treatment and recovery**. The difference in the high degree of the color is deeper, and differences in the lower level of the color is shallower. Red for rise, blue for down. Color matrix expressed the different fold in metabolite levels respectively at cold treatment 2 h and recovery 24 h between *adh1* and WT under the certain condition (CK, the control group 23°C always; S, cold shock −6°C for 2 h; A, cold acclimation 4°C for 7 d, then treated at −6°C for 2 h).

The metabolite abundance indicates changes in metabolic activity of a pathway. To assess how plant metabolism deals with cold treatments and recovers in mild temperatures (23°C for 24 h), a comparative metabolic analysis was performed for both *adh1* and WT (Figure 8).

Firstly, the change fold metabolites of WT/*adh1* were insignificant in this analysis except for sucrose in the control group, which showed decreasing levels (the background level in 23°C), suggesting high enrichment of sucrose in the *adh1* mutant. Interestingly, under freezing treatment (with or without cold acclimation), the change-fold of metabolites was significantly increased; however, after recovery for 24 h, all metabolites returned to a level consistent with the control group. The response of increasing levels of succinic acid, glutaric acid, and maleic acid were observed in cold acclimation in WT/*adh1* analysis, suggesting their enrichment was affected in *adh1* mutation, as were the decreases in the levels of tyrosine, phenylalanine, valine, asparagine, lysine, fumaric acid, and pyroglutamate during the recovery phase in the acclimation group (cold acclimation 4°C for 7 d, followed by treatment at −6°C for 2 h). The most severe recovery responses were observed for maleic acid, and following the removal of cold treatment, this strongly reduced compared to the control level, causing a corresponding change of fumaric acid.

Secondly, the changes of metabolites were detected between the freezing treatment with cold acclimation (A: cold acclimation at 4°C for 7 d, then treated at −6°C for 2 h) and cold shock without cold acclimation (S: cold shock −6°C for 2 h) in *adh1* and WT. The fold-change of metabolites of S/A in this analysis is presented in Figure 8. Almost all detected metabolites of S/A decreased in *adh1*, i.e., these metabolites accumulated more during cold acclimation than during cold shock. However, the metabolites increased only after cold treatment was removed and recovery had lasted for 24 h in *adh1* mutant seedlings. Similar results were found for WT. The only difference is that asparagine did not increase, but decreased during recovery. However, for sucrose, the fold-changes of metabolites of S/A were essentially identical for both cold acclimation group and cold shock in *adh1*, but were reduced in WT.

Furthermore, the fold-change of metabolites of A/CK in *adh1* and WT was analyzed. galactinol, melibiose, cellobiose, gentiobiose, maltose, gluconic acid, proline, and glycine levels increased both in *adh1* and WT under cold acclimation, and subsequently returned to control level or maintained the response level after removal from the cold treatment and 24 h recovery. In contrast, the fold-change of metabolites of S/CK in *adh1* and WT, beta-alanine, and glycine significantly changed from initial reduction to a dramatic increase in cold shock and recovery. However, they have been increasing in the cold acclimation group.

In summary, *ADH1* abundantly affected some metabolites, especially numerous sugars (e.g., sucrose), and amino acids (e.g., asparagine). These metabolites are intermediates of glycolysis, tricarboxylic acid cycle, and anaerobic respiration.

## Discussion

In this study, an increased expression of *ADH1* was detected in *Arabidopsis* in response to cold stress of the plant. Other *ADH2* homologs encoded in the *Arabidopsis* genome did not appear to participate in the plant's cold response under the defined experimental conditions. To investigate the physiological role of *ADH1* in cold stress response, *Arabidopsis adh1* homozygous mutants were screened from T-DNA lines, and metabolite profiling and network analyses were conducted. Metabolic profiling analyses were performed using gas chromatography-mass spectrometry to determine metabolite temporal dynamics associated with the induction of an acquired freezing tolerance in the plant in response to freezing stress with or without prior cold acclimation in both the WT and *adh1* mutants. Retention time indices and specific mass fragments were used to monitor 263 variables and annotate 78 identified metabolites. The experimental results suggested that cold stress with cold acclimation influenced the plant's metabolism far more than submitting the plant to a single cold shock experience without prior cold acclimation. However, a cold shock to a plant without cold acclimation influenced the metabolism far more than a prior cold acclimation during a 24 h recovery. In the case of cold response, WT and *adh1* reacted differently in terms of the deposition of sugars, organic acids, and amino acids. In particular, when the freeze response of WT and *adh1* were compared, sucrose was found to be the metabolite that was most sensitive to the freeze response of the two. In addition, correlation-based network analysis revealed that *adh1* exhibited distinctive coordinated stress-related changes in comparison to WT. The network analysis also highlighted that some metabolites, e.g., melibiose, fumaric acid, succinic acid, glycolic acid, and xylose, enhanced connectedness in the *adh1* network that experienced a cold shock. Therefore, when the plants experienced cold stress, *ADH1* impacted the accumulation of metabolic substances, which were involved in the plant's antifreeze reaction. This results provide new insight into the mechanism of *ADH1* response at the metabolite level during cold stress in plants and reveal a previously unknown role of *ADH1*.

*Arabidopsis* has the ability to survive below −8°C via cold acclimation (Gilmour et al., 1988). Here, semi-lethal temperatures (LT50) of *Arabidopsis* were 4°–5°C. However, the semi-lethal temperatures (LT50) of *Arabidopsis* dropped to 6°–7°C via cold acclimation (4°C for 7 d), and the *adh1* mutant revealed an increased percentage of ion leakage than WT plants after freezing treatment. This suggests that plasma membranes were more damaged in the mutant during the freezing treatment. In the response to cold stress in soybeans, the accumulation of *ADH1* and other typical cold response genes was not significant, which was considered to be the reason for poor acclimation capability and cold intolerance (Yamasaki and Randall, 2016). Environmental stresses such as hypoxia (Johnson et al., 1994), drought (Senthil-Kumar et al., 2010), and abscisic acid phytohormone (Zhang et al., 1997) induce a high expression of *ADH1*. ADH gene expression, enzyme activity, and related metabolites were analyzed under different temperatures, and the results revealed that the levels of acetaldehyde, ethanol, acetic acid, and ethyl acetate fluctuated at 10°, 20°, and 30°C (Cirilli et al., 2012), suggesting temperature affecting ADH activity and the accumulation of related metabolites. Formate levels were reduced, acetate levels were elevated, and extracellular glycerol accumulated in *adh1* mutant of *Chlamydomonas reinhardtii* under anoxic conditions (Magneschi et al., 2012). Metabolic changes are the first response to stress in plants; however, the changes of metabolic responses and their role in *adh1* mutants subjected to freezing stress remain poorly understood. The metabolic profile and response level of *adh1* mutants to different cold treatments (freezing treatments with or without prior cold acclimation) were examined in this study. Our results reveal that WT and *adh1* respond and adapt to cold stress via rapid re-establishment of metabolites. Furthermore, there was some specific accumulation of metabolites in *adh1*, such as the sucrose. Re-establishment of metabolites, especially shifts in sugars and amino acids, are the guarantee of plant survival in *adh1* mutants under freezing stress. This result demonstrates a plastic response of the metabolism in the *adh1* mutants, sustaining normal growth under optimal as well as freezing conditions.

Plastic responses have been reported for the metabolome during temperature changes (Sun et al., 2016). ROS (reactive oxygen) rather than CBF (C-repeat binding transcription factor/dehydrate responsive element binding factor) dominates rice gene expression and adaptation to low-temperatures, which is consistent with results for antioxidation and accumulation of numerous amino acids gathered via comparative metabolomic analysis (Zhang et al., 2016). Here, a significant difference between *adh1* and WT was found for the accumulation of sucrose. Transcript and metabolite profiling in *Arabidopsis* revealed a relationship between transcript and metabolic abundance during cold acclimation (Kaplan et al., 2007). Furthermore, CBF3/DREB1A will activate multiple chemical changes especially to soluble sugars (Gilmour et al., 2000). The metabolome analysis of CBF3 over-expressing seedlings revealed a prominent role of CBF in cold acclimation (Cook et al., 2004). Here, the result showed a significant difference of metabolites between freezing treatments with and without prior cold acclimation. Moreover, the change of metabolic abundance is difference between *adh1* and WT during cold acclimation or cold shock treatment, especially in sucrose and some amino acids. At the background level (optimum temperature), the abundance of sucrose in *adh1* mutant was significantly higher than in WT, which was an indication of the absence of ADH1 activity that effected the synthesis or metabolism of sucrose. Because ADH1 is the terminal enzyme of anaerobic metabolism, a feedback regulation for related pathways may be formed when the activity of ADH1 is deficient. The content of sucrose in the WT increased more significantly than in *adh1* following the freezing stress with prior cold acclimation, indicating that ADH1 can affect the response of the plant to cold acclimation. After 24 h of thermal recovery at the optimum temperature, the abundance of sucrose restored the plant to the previous state of freezing stress.

The changes of soluble sugar and amino acid accumulation in *adh1* mutants in response to freezing stress need to be further assessed, while the variability in the secondary metabolism may be further discussed in the *adh1* mutant response to freezing, since ADH1 is closely related to acetaldehyde, ethanol, and the flavonoid metabolism. The differences in the accumulation of secondary metabolites might be more pronounced between *adh1* and WT.

Due to global warming, plants may suffer from repeated freeze-thaw cycles caused by more frequent short-term low temperatures such as snow or night frost (Dolezal et al., 2016). The ADH gene is involved in the anti-freezing process of plants, and if the ADH gene was missing, membrane lipid peroxidation increased (Peters and Frenkel, 2004; Davik et al., 2013). In a previous study, the expression of *ADH1* in response to cold stress was increased in alpine subnival plants, which was affected by DNA methylation (Song et al., 2015). In the present study, to further understand the function of *ADH1* in *Arabidopsis*, the gene expression and related metabolites were analyzed in *adh1* and WT in response to short time freezing temperatures. Via analyzing the shift of metabolites, we furthered the understanding of the function of the *ADH1* gene and the adaptation strategy of plants to an increasingly variable climate.

## Author contributions

LA and YS contributed to conceive, design and coordinate the experiments. LL, GL, and YW cultured plant material and performed the experiments. YS and LL analyzed the data. XY participated to the manuscript revision. YS wrote and edited the manuscript. All authors read and approved the final manuscript.

### Conflict of interest statement

The authors declare that the research was conducted in the absence of any commercial or financial relationships that could be construed as a potential conflict of interest.

## Supplementary material

Supplementary File 1 **Metabolome.xlsx**.

Supplementary File 2 **PCALoading.xlsx**.

Supplementary File 3 **Network.xlsx**.

Figure S1 **The partial least squares-discriminate analysis (PLS-DA) of Metabolites. (A)** The score plot of PLS-DA model; **(B)** The Permutation test for the PLSDA model.

Figure S2 **Symmetric difference network based on correlation between cold treatment and metabolites**. Node colors represent different primary metabolites. Green for the amino acid, lavender for organic acid, purple for sugar, red for the nucleotide, brown for the polyol, dark green for the polyamine, blue for the other. Edges between nodes represent correlations identified as significant at |r| > 0.9, FDR < 0.05, where blue edges specific to adh1 and red edges specific to WT. **(A)** In adh1, where blue edges specific to the control group and red edges specific to cold acclimation. **(B)** In WT, where blue edges specific to the control group and red edges specific to cold acclimation. **(C)** In adh1, where blue edges specific to the control group and red edges specific to cold shock. **(D)** In WT, where blue edges specific to the control group and red edges specific to cold shock.

Table S1 **Primers for rt-PCR and qPCR**.
