# Supplementary material for: Metabolite Profiling of adh1 Mutant Response to Cold Stress in Arabidopsis
Source: Front Plant Sci. 2017 Jan 11;7:2072. doi: 10.3389/fpls.2016.02072 (PMC5225106; doi:10.3389/fpls.2016.02072)
Supplement: Figure S1 — The partial least squares-discriminate analysis (PLS-DA) of Metabolites. (A) The score plot of PLS-DA model; (B) The Permutation test for the PLSDA model. [file DataSheet4.docx]

Supplementary Material 4

**Metabolite profiling of *adh1* mutant response to cold stress in *Arabidopsis***

Yuan Song*†, Lijun Liu†, Yunzhu Wei, Gaopeng Li, Xiule Yue & Lizhe An*

***Correspondence:**

Lizhe An; Tel: +86 931 8912560; Email: lizhean@lzu.edu.cn

Yuan Song; Tel: +86 931 8912560; Email: [songyuan@lzu.edu.cn](mailto:songyuan@lzu.edu.cn)

# Supplementary Figures and Tables

## Supplementary Figures


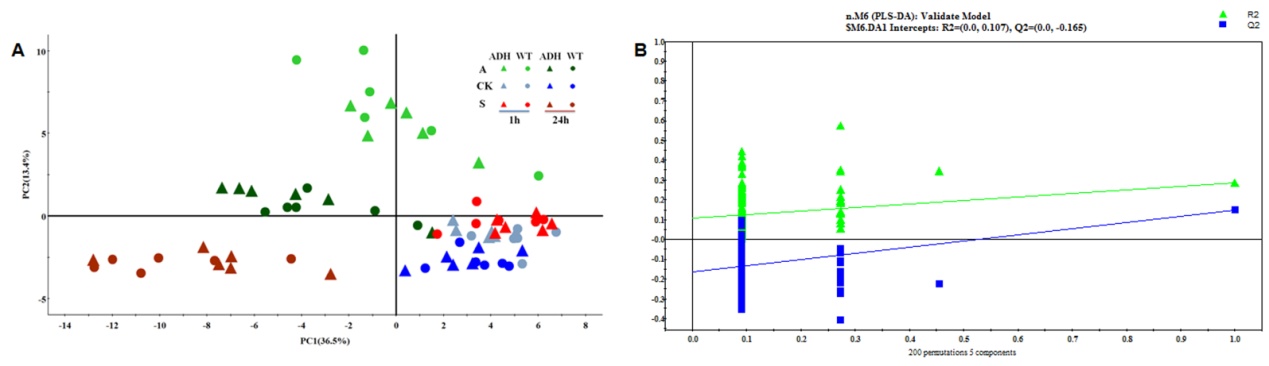


**Figure S1.** The partial least squares-discriminate analysis (PLS-DA) of Metabolites. (A) The score plot of PLS-DA model; (B) The Permutation test for the PLSDA model.


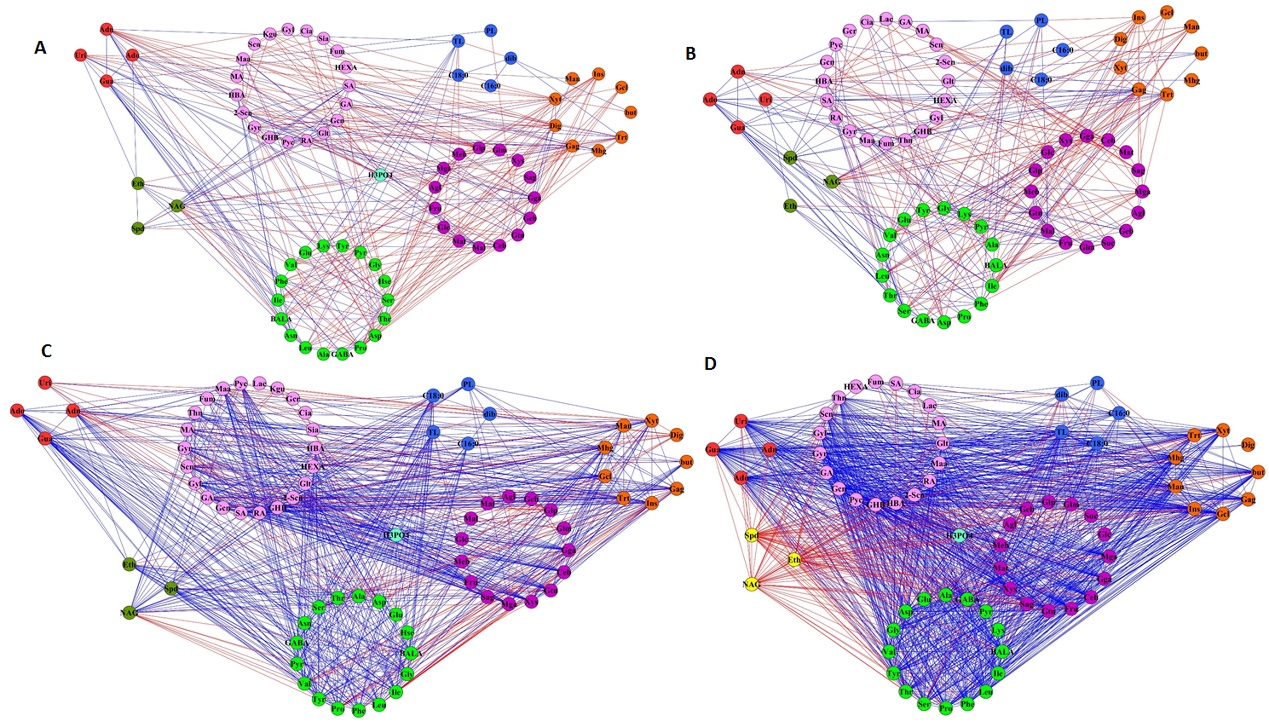


**Figure S2**. Symmetric difference network based on correlation between cold treatment and metabolites. Node colors represent different primary metabolites. Green for the amino acid, lavender for organic acid, purple for sugar, red for the nucleotide, brown for the polyol, dark green for the polyamine, blue for the other. Edges between nodes represent correlations identified as significant at |r|>0.9, FDR<0.05, where blue edges specific to adh1 and red edges specific to WT. (A) In *adh1*, where blue edges specific to the control group and red edges specific to cold acclimation. (B) In WT, where blue edges specific to the control group and red edges specific to cold acclimation. (C) In *adh1*, where blue edges specific to the control group and red edges specific to cold shock. (D) In WT, where blue edges specific to the control group and red edges specific to cold shock.

**Table S1.** Primers for rt-PCR and qPCR

| Primers | Sequence |
| --- | --- |
| RT-AtADH1-F | 5' TGATAGACAGACGCTGCTTTG 3' |
| RT-ATADH1-R | 5' CGATTGTGTTACAGTTAGTCCTAC 3' |
| qRT-AtADH1-F | 5' CCACTGATGTAGCAGCAAGG 3' |
| qRT-ATADH1-R | 5' CAGAACCAGAGGATCGGACT 3' |
| qRT-AtADH2-F | 5' GTGAGTCACACTGTTCCAATTTACG 3' |
| qRT-ATADH2-R | 5' CACCACTGAGCACAATGTTTCC 3' |
